# Projected 24-hour post-dose ocular itching scores post-treatment with olopatadine 0.7% versus 0.2%

**DOI:** 10.1007/s10928-018-9588-7

**Published:** 2018-04-21

**Authors:** Matthew L. Fidler, Abayomi Ogundele, David Covert, Ramesh Sarangapani

**Affiliations:** 1 Novartis Pharmaceutical Corporation, 6201 South Freeway, Fort Worth, TX 76134-2099 USA; 2 Alcon Laboratories, Inc., Fort Worth, TX USA

**Keywords:** Allergy, Antihistamine, Differential odds, Kinetic pharmacodynamic (KPD), Mast cell stabilizer

## Abstract

Olopatadine is an antihistamine and mast cell stabilizer used for treating allergic conjunctivitis. Olopatadine 0.7% has been recently approved for daily dosing in the US, which supersedes the previously approved 0.2% strength. The objective of this analysis was to characterize patients who have better itching relief at 24 h when taking olopatadine 0.7% treatment instead of olopatadine 0.2% (in terms of proportions of responses) and relate this to the severity of baseline itching as an indirect metric of a patient’s sensitivity to antihistamines. A differential odds model was developed using data from two conjunctival allergen challenge (CAC) studies to characterize individual-level and population-level response to ocular itching following olopatadine treatment and the data was analyzed retrospectively. This modeling analysis was designed to predict 24 h ocular itching scores and to quantify the differences in 24 h itching relief following treatment with olopatadine 0.2% versus 0.7% in patients with moderate-to-high baseline itching. A one-compartment kinetic-pharmacodynamic E_max_ model was used to determine the effect of olopatadine. Impact of baseline itching severity, vehicle effect and the drug effect on the overall itching scores post-treatment were explicitly incorporated in the model. The model quantified trends observed in the clinical data with regards to both mean scores and the proportions of patients responding to olopatadine treatment. The model predicts a higher proportion of patients in the olopatadine 0.7% versus 0.2% group will experience relief within 24 h. This prediction was confirmed with retrospective clinical data analysis. The number of allergy patients relieved with olopatadine 0.7% increased with higher baseline itching severity scores, when compared to olopatadine 0.2%.

## Introduction

Allergic conjunctivitis is a common form of ocular allergy caused by immunoglobulin E-mediated inflammatory reaction to an allergen [1, 2]. The prevalence of allergic conjunctivitis ranges from 15 to 40%, depending on geographic location and patients’ age [3]. A majority of these cases are attributed to seasonal allergic conjunctivitis or perennial allergic conjunctivitis [4]. Allergic conjunctivitis is associated with significant economic and healthcare burden and reduction in both ocular quality and general quality of life [5–7]. In addition, it may lead to work quality and productivity impairment [8–10]. Various treatment options are available for allergic conjunctivitis, these include antihistamines, mast cell stabilizers or nonsteroidal anti-inflammatory drugs [11]. Olopatadine is an antihistamine and mast cell stabilizer used for treating allergic conjunctivitis [12]. Olopatadine hydrochloride 0.1 and 0.2% strengths were approved by the US Food and Drug Administration in 1996 and 2004 respectively [13]. Olopatadine 0.7% was approved as a once-daily topical ocular treatment for ocular itching associated with allergic conjunctivitis in the US in 2015 [14]. In clinical studies, the high concentration of olopatadine showed superior relief from itching, 24 h after dosing compared to the previously approved 0.1 and 0.2% olopatadine formulations [15, 16].

A survey was performed in 149 patients receiving olopatadine 0.2% to assess their beliefs and prescribing practices for the current PATADAY™ product [17]. This study concluded that a high dose may be useful in these patients. The main findings of this study were: (1) some patients felt the 0.2% dose lasted for 9 h; (2) one-third of the patients did not feel their symptoms were completely resolved and used more than 1 product for additional relief; (3) 38% patients were recommended to use the product twice a day; (4) 30% patients used the medication twice daily; and (5) 26% patients varied their daily dose depending on symptom severity, with 50% patients more likely to exceed the recommended dose. It is likely less patients will require more than 1 prescription of olopatadine 0.7% per month than if olopatadine 0.2% was prescribed, leading to better compliance and relief [15, 16].

The efficacy of olopatadine 0.2% can be determined on the basis of the PATADAY™ (olopatadine 0.2%) prescription database study [17]. This study included 170,000 patients who had received olopatadine 0.2% at least once a year, as obtained from the prescription claims from Market Scan (April 2008–March 2013). This database showed that, annually, 31% patients require 2–3 prescriptions and 14% require 4 or more (range 4–23) prescriptions. This study suggested that a subgroup of patients require more antihistamine relief than what is provided by a daily single dose of olopatadine 0.2%. Two conjunctival allergen challenge (CAC) trials (C-10-126 or NCT01479374 and C-12-053 or NCT01743027) demonstrated that olopatadine 0.7% dose at 24 h prior to allergy challenge provides clinically superior itching relief to olopatadine 0.2% [15, 16]. However, this study was not designed to look at clinical response in patients who had insufficient itching relief on olopatadine 0.2% or to quantify the number of these patients who would be relieved with olopatadine 0.7%. This question can be answered by a model-based bridging analysis. Therefore, a model was created to simulate and predict patients with high itching scores in CAC trials who would require more antihistamine relief than that provided by olopatadine 0.2%.

While the 2 prior clinical studies showed improved 24-h control, the survey data showed that there is a subset of patients who are more sensitive to ocular allergies and were insufficiently controlled on olopatadine 0.2%. The prior clinical trial was not conducted to assess the itching response in these more sensitive patients, but their response is important to capture. For this reason, a mathematical model was developed to quantify the itching severity in more severe patients. The objective of this analysis was to apply a qualified model to determine the patients who had better itching relief at 24 h when taking olopatadine 0.7% treatment instead of olopatadine 0.2%, in terms of proportions, and relate this to the severity of baseline itching as an indirect metric of a patient’s sensitivity to antihistamines.

## Methods

### Clinical data summary

The data used in this analysis were obtained from the intent-to-treat (ITT) datasets derived from 2 CAC trials: C-10-126 (NCT01479374) and C-12-053 (NCT01743027).

C-10-126 was a US multicenter, double-masked, parallel-group study of adult patients with a history of allergic conjunctivitis who were randomized to vehicle (68 patients), 0.2% olopatadine (68 patients, 66 ITT patients), or 0.7% olopatadine (66 patients) in both the eyes [16]. Patients were required to have a positive bilateral CAC response to an allergen, which was based on individual patient allergic sensitivity at visit 1 (day 21) and visit 2 (day 14), during the screening period. A positive response at visit 1 (day 21) was defined as an itching score of ≥ 2 units for each eye and a redness score of ≥ 2 units in 2 of the 3 vessel beds (ciliary, conjunctival, or episcleral) within 10 min of the last titration challenge. At visit 2, which was 1 week later, a positive response was defined as an itching score of ≥ 2 units for each eye and a redness score of ≥ 2 units in 2 of 3 vessel beds for at least 2 of the 3 post-CAC time points. Visit 2 (day 14) onwards, itching was assessed at 3-, 5-, and 7-min post-allergen challenge. Patient-reported ocular itching was scored from 0 (no itching) to 4 (incapacitating itch) in 0.5 unit increments (18). After the 2-week screening period, patients were randomized to receive either olopatadine 0.2 or 0.7%. CAC with the same amount of allergen that was titrated to at screening was administered 24-h post-drug administration, and ocular itching was assessed at 3, 5 and 7 min post challenge for both eyes. After 2 weeks, first dose of the drug was administered (one drop of the drug was bilaterally instilled to each eye), and 16-h post-drug instillation, ocular itching CAC with the titrated level of allergen was given to the patient. Itching scores were then recorded at 3, 5 and 7 min post-challenge for both eyes. Another dose was administered the next week and ocular CAC challenge was administered 27-min post-dose instillation (the onset of action; itching was measured 3, 5, and 7 min post-challenge) [16]. The 16 and 24 h time-points were used to compare the trough effect after once-a-day dosing (24 h) and twice a day dosing (16 h) of olopatadine. The primary efficacy endpoint was itching relief superiority of olopatadine 0.7% over vehicle at the onset of action and 16-h post-dose. The patients receiving olopatadine 0.7% showed significantly better response to primary and secondary endpoints (*p *< 0.05). Additionally, the responses of patients 24-h after olopatadine 0.7% were significantly different from that of those receiving the vehicle. A summary of these responses is shown in Table 1. C-12-053 was a US multicenter, double-masked, parallel-group study in adult patients with a history of allergic conjunctivitis [15]. Patients were randomized to vehicle (49 patients), 0.1% olopatadine (99 patients), 0.2% olopatadine (99 patients), or 0.7% olopatadine (98 patients) in both the eyes. Patients were screened in the same manner as that in C-10-126. CACs were separated by 2 weeks. The first challenge tested ocular itching 24-h post-instillation, and the second challenge tested ocular itching 27-min post-instillation (onset of action) [15]. The primary efficacy endpoint was to compare itching scores with olopatadine 0.7% to those with the vehicle, olopatadine 0.1, and 0.2%, at 24-h post CAC, and to check the superiority of olopatadine 0.7% compared to the vehicle at the onset of action CAC. Most time points were significant (*p *< 0.05). The differences in means are presented in Table 2.

**Table 1:** Summary of key endpoints of patients receiving olopatadine 0.7% in the registration trial C-10-126: Mean difference with 95% Confidence Intervals

	Minutes after CAC		
	3	5	7
Differences in the CAC mean itching score after the onset of action with olopatadine 0.7% versus vehicle	− 1.52** (− 1.85, − 1.20)	− 1.51** (− 1.85, − 1.18)	− 1.48** (− 1.80, − 1.16)
Differences in 16-h CAC mean itching score after olopatadine 0.7% versus vehicle	−1.50** (− 1.78, − 1.22)	−1.47** (− 1.77, − 1.17)	−1.38** (− 1.70, − 1.06)
Differences in 24-h CAC mean itching score after olopatadine 0.7% versus vehicle	−1.58** (− 1.85, − 1.31)	−1.48** (− 1.77, − 1.18)	−1.38** (− 1.67, − 1.09)
Differences in 24-h CAC mean itching score after olopatadine 0.7% versus 0.2%	−0.47* (− 0.76, − 0.19)	−0.39* (− 0.71, − 0.09)	−0.38* (− 0.70, − 0.07)

Sample size in C-10-126 study: Olopatadine 0.7% (n = 66), Olopatadine 0.2% (n = 66) & Vehicle (n = 68)

Pairwise tests at each post-challenge time point were based upon the least squares means derived from a statistical model that accounted for within patient correlated measurements

*CAC* conjunctival allergen challenge

**p < 0.0001

*p < 0.05

**Table 2:** Summary of the primary endpoints in patients receiving olopatadine 0.7% in the registration trial C-12-053: Mean difference with 95% Confidence Intervals

	Minutes after CAC		
	3	5	7
Differences in the CAC mean itching score difference after the onset of action after olopatadine 0.7% versus vehicle	− 1.53** (− 1.76, − 1.30)	− 1.46** (− 1.71, − 1.22)	− 1.17** (− 1.45, − 0.90)
Differences in 24-h CAC mean itching score after olopatadine 0.7% and vehicle	− 1.29** (− 1.60, − 0.97)	− 1.15** (− 1.46, − 0.84)	− 0.89** (− 1.22, − 0.57)
Differences in 24-h CAC mean itching score after olopatadine 0.7% and 0.1%	− 0.52** (− 0.78, − 0.27)	− 0.48* (− 0.73, − 0.23)	− 0.39* (− 0.65, − 0.12)
Differences in 24-h CAC mean itching score after olopatadine 0.7% and 0.2%	− 0.31* (− 0.57, − 0.06)	− 0.26* (− 0.51, − 0.01)	− 0.16 (− 0.42, 0.11)

Sample size in C-12-053 study: Olopatadine 0.7% (n = 98), Olopatadine 0.2% (n = 99) & Vehicle (n = 49)

Pairwise tests at each post-challenge time point were based upon the least squares means derived from a statistical model that accounted for within patient correlated measurements

*CAC* conjunctival allergen challenge

**p < 0.0001

*p < 0.05

Overall, 547 patients with 10,759 itching observations from these two CACs studies were used for the modeling analysis. These data included baseline (baseline observations 3269), and post-treatment observations: vehicle (patients, 117; observations, 1734), 0.1% (patients, 99; observations, 1134), 0.2% (patients, 167; observations, 2322), or 0.7% (patients, 164; observations, 2300). The range of total ocular itching scores per patient varied from 12 to 24. The mean (median) numbers of total ocular itching scores per patient were 19.74 (18.0). The key demographic characteristics of the patients are summarized in Table 3.

**Table 3:** Demographics by treatment for the analysis dataset (intention to treat)

	Vehicle	0.10%	0.20%	0.70%	Overall
N	117	99	165	164	545
Age (years)					
Mean (SD)	41.7 (12.8)	41.0 (12.2)	41.4 (13.9)	39.7 (12.9)	40.9 (13.0)
Median (Min, Max)	42.0 (18.0, 77.0)	41.0 (18.0, 72.0)	41.0 (18.0, 75.0)	40.0 (18.0, 68.0)	41.0 (18.0, 77.0)
Baseline itching					
Mean (SD)	2.8 (0.5)	2.9 (0.4)	2.8 (0.5)	2.9 (0.5)	2.8 (0.5)
Median (Min, Max)	2.8 (1.8, 4.0)	2.9 (2.0, 4.0)	2.8 (1.8, 4.0)	2.8 (1.5, 4.0)	2.8 (1.5, 4.0)
Gender, n (%)					
Men	46 (39.3)	43 (43.4)	68 (41.2)	60 (36.6)	217 (39.8)
Women	71 (60.7)	56 (56.6)	97 (58.8)	104 (63.4)	328 (60.2)
Allergen, n (%)					
Cat dander	13 (11.1)	5 (5.1)	11 (6.7)	17 (10.4)	46 (8.4)
Cockroach	2 (1.7)	1 (1.0)	2 (1.2)	2 (1.2)	7 (1.3)
Dog dander	1 (0.9)	1 (1.0)	4 (2.4)	2 (1.2)	8 (1.5)
Dust mites	19 (16.2)	17 (17.2)	35 (21.2)	24 (14.6)	95 (17.4)
Grass	45 (38.5)	45 (45.5)	73 (44.2)	66 (40.2)	229 (42.0)
Ragweed	27 (23.1)	15 (15.2)	16 (9.7)	32 (19.5)	90 (16.5)
Trees	10 (8.5)	15 (15.2)	24 (14.5)	21 (12.8)	70 (12.8)

The baseline measurements are at screening time only

*SD* standard deviation

### Mathematical model for itching score

**Fig. 1:**
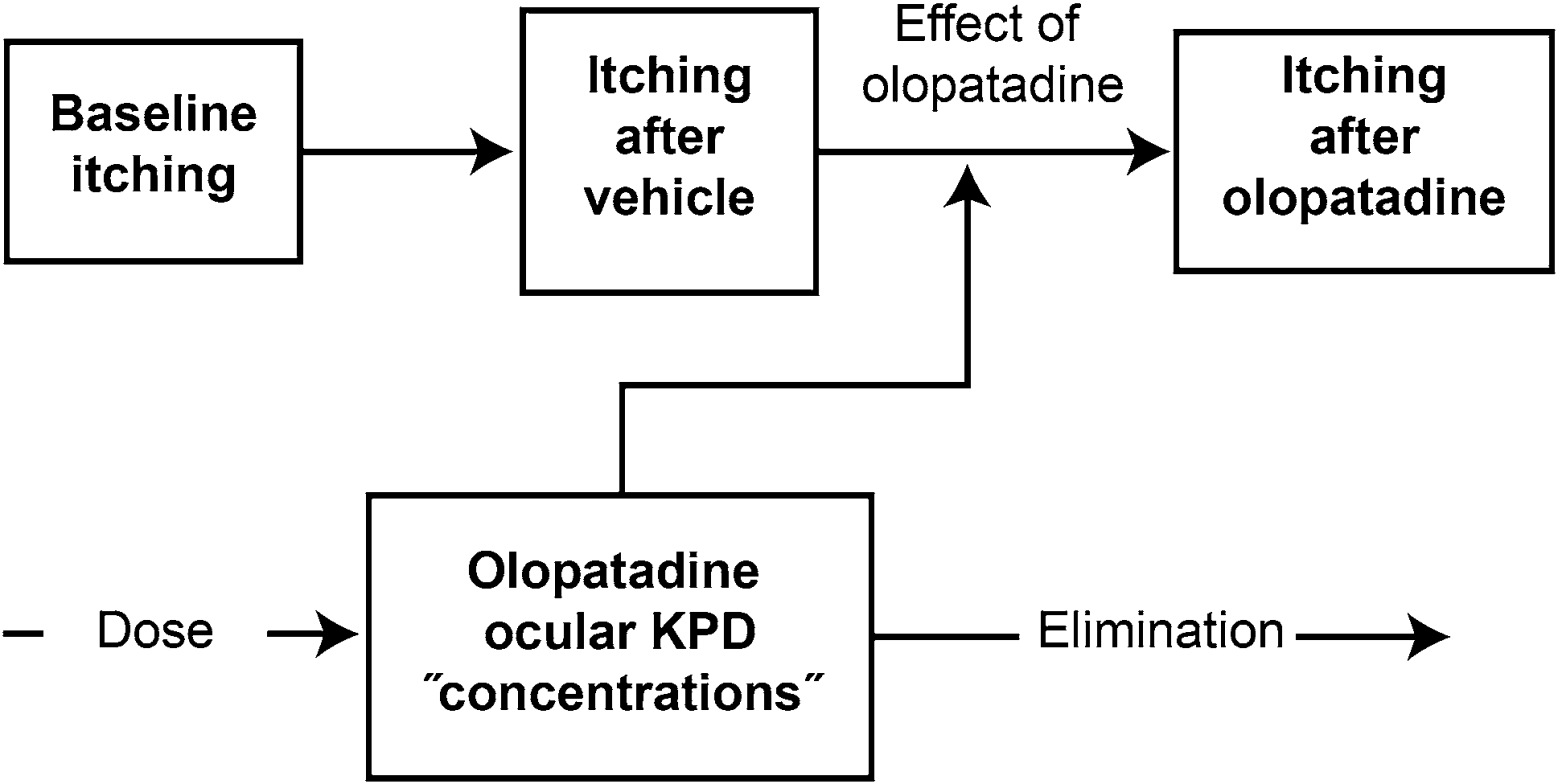
Box diagram of olopatadine model structure. *KPD* kinetic pharmacodynamic

This model was developed to describe individual-level and population-level response on ocular itching scores for various dose strengths of olopatadine, above-and-beyond the vehicle effect (Fig. 1). Itching score was used as a categorical variable in a differential odds model, instead of the mean itching score [19]. This allows the probability of each response to be modeled and outcomes to be based on the categories instead of the mean tendencies of a simple mean-response model. The differential odds model was used because it is more flexible in describing each patient’s response. Additionally, if the proportional odds model is better at describing the given data, the differential odds model will reduce to the proportional odds model. Additionally, this model parametrization allowed for comparison of the proportion of the population that would better respond to olopatadine 0.7% than to olopatadine 0.2%, which cannot be addressed by modeling mean itching score alone (as in the clinical trial).

Elimination of the theoretical olopatadine concentration from the ocular space was driven by the observed effect (CAC itching score probabilities), rather than by any observed ocular olopatadine concentrations (ocular one-compartment kinetic pharmacodynamic [KPD] “concentrations” in Fig. 1). These effects and the subsequent KPD elimination was based on time-points where CAC challenges were administered at baseline, after vehicle response and after the 27 min, 16 and 24 h itching challenges; At each of these overall time-points the itching score was measured at 3, 5, and 7 min post-challenge. Note this model is only concerned with the probability of an itching score, and not the redness response so will not give a “positive” response as defined in the study. However, the model can be used to simulate the probability of having the same itching response as a “positive” response as defined by the studies.

These observed effects on itching were parameterized and qualified using data from the two completed CAC trials described in the previous section. The model was developed in NONMEM application version 7.3 using a slow numerical conditional Laplacian estimation. Baseline and vehicle itching score probabilities were characterized with separate differential odds. A linear vehicle relationship with time was used to assess the extent of the effect of a vehicle. The olopatadine effect was characterized using a one-compartment KPD *E*_*max*_ model.

If Y_i_ = (Y_i1_,Y_i2_,…,Y_iN_) represents categorical itching scores for the *i*th individual, with *N* observations, then the probability that some observation of the *k*th observation for individual *i* Y_*ik*_ is an itching score greater than or equal to *s* has the following general structure:

$${\text{Logit}}\left[ {P\left( {Y_{ik} \ge s|\eta_{i} } \right)} \right] = f_{s} + \eta^{*}_{i} ,\quad s = \, 0.5, \, 1, \, 1.5, \ldots ,3.5, \, 4$$

Or,

$$P\left( {Y_{ik} \ge s|\eta_{i} } \right) = 1/\left( {1 + \exp \left[ { - f_{s} + \eta_{i} } \right]} \right){\text{ where}}\,\eta_{i} = - \eta^{*}_{i}$$

Individual differences in overall probabilities were modeled; *η*_*i*_ is an individual random deviation from the population probabilities. *η*_*i*_ is normally distributed with mean 0 and variance ω^2^. The *f*_s_ function is a function of baseline conditions and different predictors:

$$f_{s} = f\left( {{\text{Drug}}\,{\text{or}}\,{\text{Vehicle}}\,{\text{Effect}}} \right) + \sum\limits_{{{\text{t}} = 0.5}}^{S} {\alpha_{i} = D_{e} \left( S \right) + V_{e} \left( S \right)} + \sum\limits_{{{\text{t}} = 0.5}}^{S} {\alpha_{i} } \quad {\text{for}}\,{\text{i}} = 0.5\,{\text{to}}\,4\,{\text{by}}\,0.5$$

where baseline odds parameters are described by the baseline logit score sum parameters (α_i_). The *f* function for score *I *=0.5–4 is parameterized in two different functions: the drug effect (*D*_*e*_) and the vehicle effect (*V*_*e*_):

$$D_{e} \left( S \right) = \frac{{E_{m} C\left( t \right)}}{{EC_{50} + C\left( t \right)}} \times \left\{ {\begin{array}{*{20}c} {\prod\nolimits_{m = 1}^{S} {\delta_{m} } } & {S \ge 1} \\ 1 & {S = 0.5} \\ \end{array} } \right.$$

$$V_{e} \left( S \right) = V_{i} \left( {V_{b} + V_{m} t} \right) \times \left\{ {\begin{array}{*{20}c} {\prod\nolimits_{m = 1}^{S} {v_{m} } } & {S \ge 1} \\ 1 & {S = 0.5} \\ \end{array} } \right.$$

where *E*_*m*_ represents the maximum olopatadine drug effect, *EC*_50_ represents the KPD concentration where 50% of the maximum olopatadine effect occurs, and *C*(*t*) represents the KPD concentration () for a dose with an apparent half-life of *t*_12_.

The *δ*_*i*_ parameters are the differential odds parameters for drug effect. Additionally, the parameter *V*_*i*_ is the vehicle indictor where 0 for baseline observations, and 1 with drug or vehicle treatment. The *V*_*b*_ is the baseline vehicle effect at time zero, and *V*_*m*_ is the slope of the vehicle change in effect with respect to time *t*.

During base model development, when the covariance step did not complete successfully, cumulative log-odds or logit-transformed differential odds adjustment parameters of > 10 or < − 10 were fixed to 10 (when positive) and − 10 (when negative). A value of > 10 implied no additional change to the odds of a score, whereas that of − 10 (when negative) implied the odds for this and larger scores approach zero. Note that the score for drug response for scores 3.5 and 4 the parameters were fixed to − 10 and 10, respectively. This is because the model was closet to these values on an unconstrained estimation. However, both the probability of scoring either 3.5 or 4 when using olopatadine are both are close to zero. The -10 value that was fixed for the 3.5 score states that this score and any higher scores are close to zero; The 10 value says that changing the scale higher 4 didn’t change any of the probabilities; This is still consistent, though it may be a bit counter-intuitive. In addition, the EC_50_ was fixed to the estimated value to allow the covariance step to complete. This model was then used to evaluate covariates.

### Covariate analysis

The continuous covariate effect for a model parameter, θ, was modeled using the equation below:

$$\theta_{t} = \theta_{pop} \left( {\frac{{{\text{Individual}}\,{\text{Covariate}}\,{\text{Value}}}}{{{\text{Median}}\,{\text{Covariate}}}}} \right)^{{\psi \text{cov} }}$$

This power model was the only model used in covariate analysis of continuous covariates of population parameters (θ_s_).

For the only categorical covariate effect, gender, the following equation was used:

$$\theta_{t} = \theta_{pop} + \psi_{F} I_{F} \;{\text{where}}\,I_{F} = \left\{ {\begin{array}{*{20}c} 1 & {\text{Female}} \\ 0 & {\text{Male}} \\ \end{array} } \right.$$

Additionally, using covariates, the overall cumulative distribution function was changed. In such cases, the distribution shifted, as follows:

$${\text{Logit}}\left[ {P\left( {Y \ge S\left| {\eta_{i} } \right.} \right)} \right] = f_{S} + \psi_{\text{cov}} \left( {{\text{Individual}}\,{\text{Covariate}}\,{\text{Value}} - \,{\text{Median}}\,{\text{Covariate}}} \right) + \eta_{i}$$

This linear shift was the only functional form used in covariate analysis of the distribution shape.

For gender, the individual covariate value is the female indicator, *I*_*F*_, with the median set to 0.

Since the baseline value has a large influence on the distribution, a baseline covariate effect on the distribution was included in the base model. This baseline covariate effect was the average of all of the baseline measurements for the eye and used as a surrogate of a patient’s pollen sensitivity. After baseline, age and gender covariate effects were tested on the *E*_*max*_, and Vehicle (*V*_*b*_) parameters. Age and gender covariate effects were tested on the overall distribution. A covariate was considered significant if it lowered the objective function by 3.84 units, (*p* = 0.05 because the difference in distributions is an approximate χ^2^ distribution). Note that the covariates could affect both the drug-effect parameters and the distribution themselves. This is similar to how a covariate effect affects both the magnitude of response and the variance of the response in a proportional error model in a purely PK model. Like in the proportional PK model, this could possibly distort the “true” relationship if another distribution is correct (say a Poisson distribution or a lognormal distribution) relationship fits better; however, qualification of observations allow the model to show it predicts the data well and could be used for extrapolation in the future. The overall covariate analysis is summarized in Table 4.

**Table 4:** Univariate and olopatadine 0.7% covariate analysis

Covariate	OFV	Δ OFV	Covariate effect	Included in the final model
Univariate covariate analysis				
Base model	32,146.69			
Baseline on vehicle (V_b_)	31,185.51	− 961.18	2.27	Yes
Baseline on max effect (E_max_)	32,370.59	223.90	0.788	No
Age on vehicle (V_b_)	32,899.04	752.35	0.109	No
Age on max effect (E_max_)	32,898.80	752.11	0.109	No
Age on distribution	32,890.55	743.86	− 0.000414	No
Gender on vehicle (V_b_)	32,885.63	738.94	− 0.168	No
Gender on max effect (E_max_)	33,216.22	1069.53	0.198	No
Gender on distribution	3,2890.31	743.61	− 0.0591	No
Olopatadine 0.7% covariate analysis				
Base model	31,181.37			
Olopatadine 0.7% baseline on max effect (E_max_)	31,471.64	290.27	0.109	No
Olopatadine 0.7% baseline on distribution	31,167.23	− 14.14	− 0.64	Yes
Olopatadine 0.7% baseline on vehicle (V_b_)	31,171.39	− 9.98	0.263	No
Olopatadine 0.7% baseline on distribution and vehicle (V_b_)	31,167.41	− 13.96	–	No

*OFV* objective function value

After one round, the initial covariate screening process was completed, since all other covariates increased the objective function, the baseline’s effect on vehicle was added as the significant covariate. After this point, the EC_50_ parameter was unfixed and estimated and model qualification was run.

The model was qualified using three different clinical outcome metrics: (1) the expected mean itching score for each group (pooled baseline, vehicle, olopatadine 0.1, 0.2, and 0.7%, at onset, 16-h post-dose, or 24-h post-dose), (2) the expected percentage of patients who experienced itching relief within 24-h (a score of < 1.0 or < 1.5 at 24-h post-dose), and (3) the proportion of patients in whom the allergy was relieved at 24-h using 0.7% olopatadine compared to that with 0.2% olopatadine. After the first round of covariate selection, the selected model qualified the first two metrics, but failed the last clinical outcome metric. Since the third qualification step focused on the olopatadine 0.7% response, covariate effects specific to olopatadine 0.7% dose were included in the covariate model (Table 4). Upon two rounds of selection, the best model described the average baseline having an additional shift in the probability distribution for the 0.7% dose, implying an even greater effect than expected with the 0.7% dose. This updated model was the best final model based on all the goodness-of-fit qualification steps. These qualification steps compared the simulated mean and variability to the observed mean and variability either graphically or numerically, and are described below.

### Model qualification

This model was built on the probability of each patient realizing an itching score after an allergen challenge. However, the mean itching score per time-point and treatment and expected percent of patients with itching relief 24-h post-dose were not included in the model structure. Therefore, simulating both the mean itching score and percentage of patients with 24-h relief and comparing this result with the observed CAC data will qualify that the model is reasonable and can predict the observed mean differences in 24-h itching relief of olopatadine 0.2% and olopatadine 0.7%. Note the observed mean differences do not imply that any one particular score is predicted well, but rather the mean of all the scores are similar to what is observed in the study and can be used to predict mean responses in other studies.

### Population Simulation

Following model qualification, model-based simulation was used to predict the differences in 24 h itching relief with olopatadine 0.7% versus 0.2%. These simulations were performed as a function of baseline itching scores as a surrogate measure of histamine sensitivity. The modeling analysis was used to quantify how many more patients would have relief on olopatadine 0.7% than with olopatadine 0.2%.

The model simulated the proportion of patients who achieved itching relief with olopatadine 0.2 or 0.7% within 24 h in the general population. In the CAC trials, baseline itching was measured in both eyes at 3-, 5-, and 7-min post-allergen challenge, making 6 itching observations per baseline screening (3/eye). Multiple scenarios were tested; the first variable changed was baseline severities; these were screened so that 1/6–5/6 of the total individual itching observations at baseline would have either itching scores of ≥ 2, ≥ 2.5, ≥ 3, or ≥ 3.5 to represent the population with moderate-to-severe itching. To account for uncertainty in parameter estimates and to obtain a better estimate of the general population’s outcome, the population-based parameters, like the overall baseline itching probabilities, were sampled based on their uncertainty to create a virtual cohort.

Patient baseline characteristics were simulated by sub-setting observed CAC study baselines that satisfied the desired eligibility criteria, and by assuming that the proportions of patients with a baseline CAC study are the same as those observed in a new simulated study. Because the CAC baselines were observed at 3- 5- and 7- min post-challenge, the simulation also produced baselines itching scores for both eyes at 3-, 5- and 7- min post-challenge.

For each of the 8 enrollment criteria (i.e. 2/6 eyes ≥ 3.5 itching score), one hundred studies were simulated. Each study used the uncertainty in the fixed effects model parameters to have slightly different study characteristics and account for uncertainty in the estimated model parameters. For each of the 100 simulated studies, 100 patients were enrolled for each treatment and their 24 h itching score was recorded. This captured the overall population and provided a quantitative estimate of the differences in itching resolution in the virtual patient population with different itching sensitivities.

## Results

The model structure is shown in Fig. 1, and the final model parameters are shown in Table 5.

**Table 5:** Final parameter estimates

Score	α_s_, Baseline cumulative log-odds estimate (RSE %)	v_s_, Vehicle differential log-odds estimate (RSE %)	δ_s_, Drug differential log-odds estimate (RSE %)
Log & differential odds parameters			
0.5	8.79 (2.50)		
1	− 1.47 (4.22)	10.00 (FIXED)	10.00 (FIXED)
1.5	− 1.03 (4.61)	10.00 (FIXED)	10.00 (FIXED)
2	− 0.85 (12.34)	10.00 (FIXED)	3.64 (48.17)
2.5	− 1.88 (5.61)	2.38 (11.27)	1.56 (17.60)
3	− 2.88 (4.60)	1.07 (11.90)	10.00 (FIXED)
3.5	− 3.02 (3.82)	10.00 (FIXED)	− 10.00 (FIXED)
4	− 2.14 (7.33)	10.00 (FIXED)	10.00 (FIXED)
Vehicle parameters			
Parameter	Estimate (%RSE)		
Vehicle linear shift (V_b_)	− 4.98 (5.65)		
Vehicle time shift (V_m_)	0.06 (14.07)		
ψ_baseline on Vb_	2.16 (5.04)		
Olopatadine parameters			
Parameter	Estimate (%RSE)		
KPD drug half-life (t_1/2_, h)	18.46 (98.37)		
EC_50_ (%)	0.03 (96.36)		
Maximum effect, E_max_	3.40 (8.70)		
Distribution parameters			
Parameter	Estimate (%RSE)		
ψ_baseline, distribution_	5.37 (3.96)		
ψ _Olopatadine 0.7 % shift on baseline, distribution_	− 0.64 (54.17)		
Log odds SD (w)	1.38 (4.76)		

With the exception of half-life and EC50, all parameters relate to the log-odds probability of having a score from 0 to 4, and are unit less like probability measurements. Half-life is measured in terms of hours, and EC50 is measured in terms of theoretical concentration based on dose, and the units for EC50 are unknown, though could match the dose (which was measured in %)

*KPD* kinetic pharmacodynamic, *RSE* relative standard error, *SD* standard deviation

**Fig. 2:**
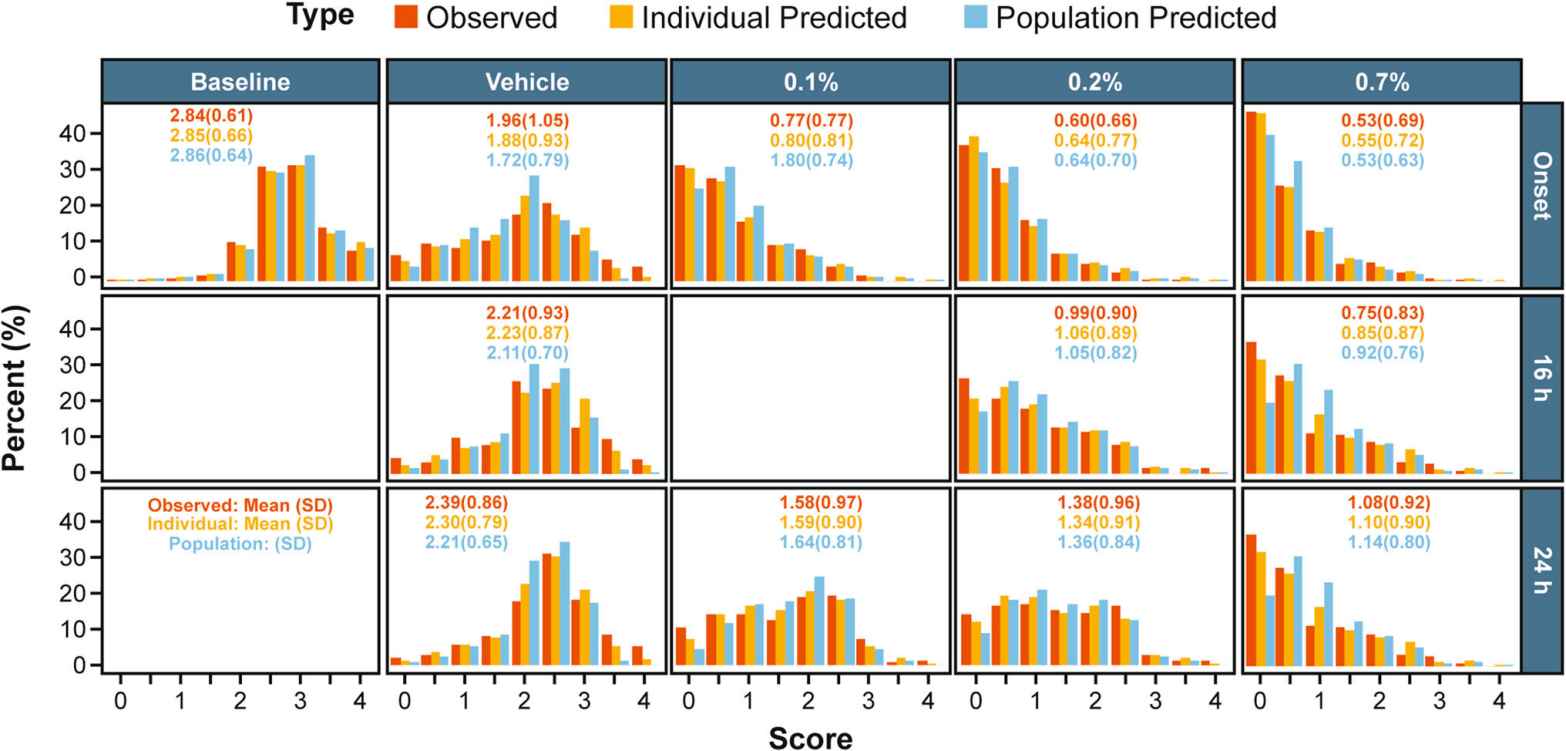
Histogram of observed, individual predicted, and population predicted itching score frequencies, stratified by treatment and nominal time-point. This figure shows a simulation of treatment (baseline, vehicle, 0.1, 0.2 and 0.7%) and the time-points of CAC (Onset, 16 h, and 24 h). The 3, 5, and 7 min post-CAC itching measurements were simulated for 100,000 subjects. Two different types of simulation were performed, with and without between subject variability. Each category’s simulated itching proportion is graphically compared to the observed itching proportions. Additionally, the plot is annotated with the mean (SD) for the itching scores under the simulation conditions and observed between the pooled clinical studies. Overall, population predictions are less precise than the individual predictions, and vehicle/baseline predictions are more precise than predictions in the presence of olopatadine. Additionally, there seems to be a slight under-prediction of the 0.7% doses’ ability to produce no-itching at any time-point, but more especially the later time-points. However, the other scores seem to be predicted fairly well. *CAC* conjunctival allergen challenge, *SD* standard deviation

Figure 2 shows the observed proportions and simulations of treatment (baseline, vehicle, 0.1, 0.2 and 0.7%) and the various time-points of CAC (Onset, 16 and 24 h). The 3, 5, and 7 min post-CAC itching measurements were simulated for 100,000 subjects. Two different types of simulation were performed, with and without between subject variability. Each category’s simulated itching percent is graphically compared to the observed itching percentages; additionally, the plot is annotated with the mean (SD) for the itching scores under the simulation conditions and observed between the pooled clinical studies. Overall, population predictions are less precise than the individual predictions, and vehicle/baseline predictions are more precise than predictions in the presence of olopatadine. Additionally, there seems to be a slight under-prediction of the 0.7% doses’ ability to produce no-itching at any time-point, but more especially the later time-points; however the other scores seem to be predicted fairly well. Additionally, the probabilities seem descriptive enough to produce reasonable estimates for mean responses for all the categories, even though the mean responses were not directly used for the model.

**Fig. 3:**
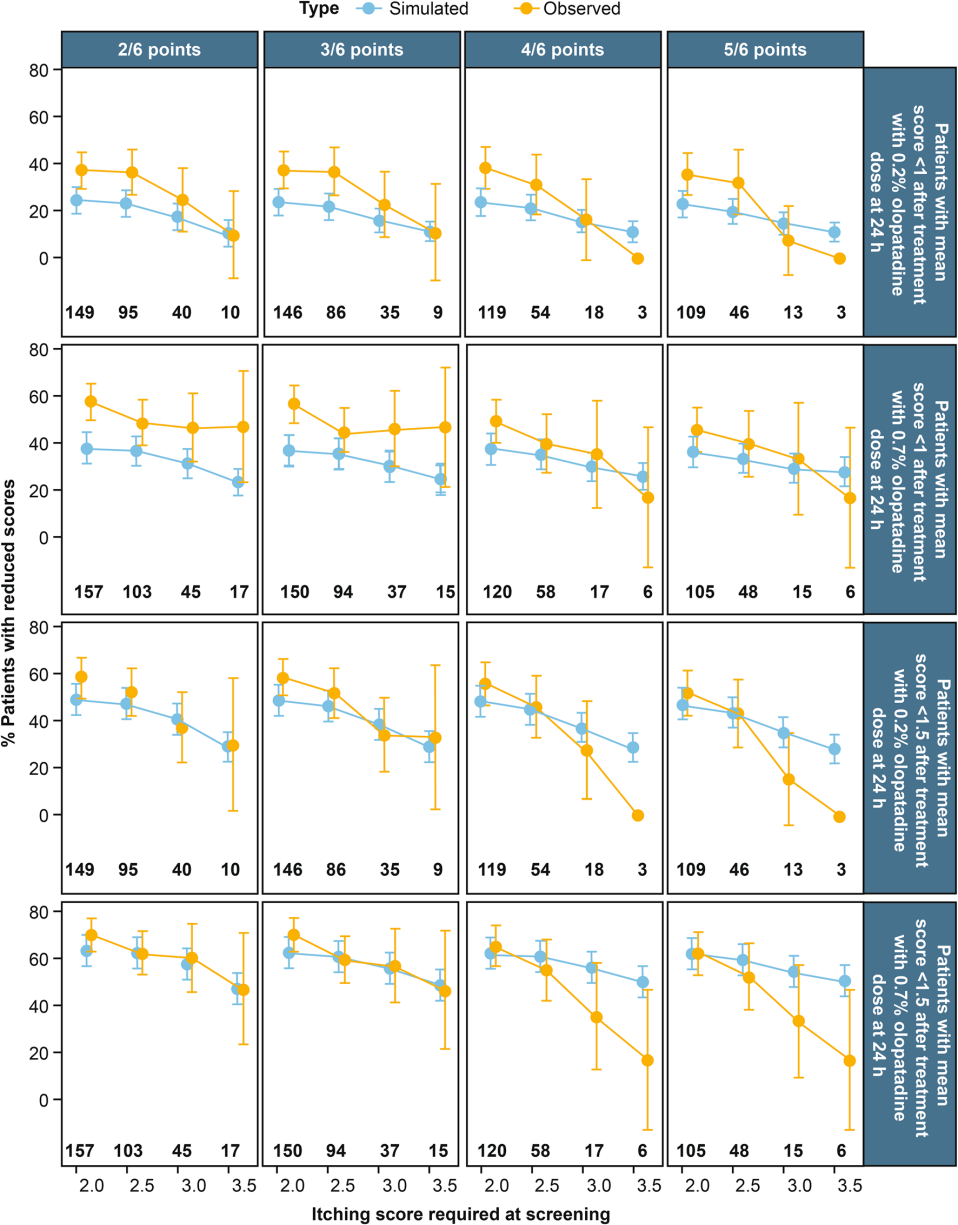
Percentage of patients adequately controlled at 24 h post-dose when administered olopatadine based on itching score screening criterion (2/6–5/6 observations ≥ of 2–3.5 itching score). The x-axis on this figure shows the screening score that is required for patients to be enrolled in the study. The columns of the figure stratify the study design to have from 2/6 to 5/6 observations needed to be enrolled in the study. The columns are stratified to patients who achieve adequate 24-h relief with the 0.2 and 0.7% olopatadine doses and with two different definitions of adequate relief (mean itching score of all observed times < 1 at the top and < 1.5 at the bottom). The doses are compared in the first two rows for < 1 itching score having adequate relief. For the 0.7% dose the initial study designs ~ 40% of the population with adequate relief, while the 0.2% have ~ 25% of the population with adequate relief. The shallow slope of the curves show that the 0.7% dose does not change its effect as much as the slightly sharper decline in the 0.2% dose. However, this change in slope difference should be interpreted with caution since it is based on sparse data at the more stringent and may be changed with more data. This trend is also repeated in the bottom two rows, with closer alignment of the data with the simulated trends. This difference in predictive power is likely due to the poorer performance of the no-itching response in the 0.7% dose that was shown in Fig. 2. Regardless, the data is suggestive of more relief from the higher dose in a larger proportion of patients than with the 0.2% dose. Numbers on the bottom represent the number of observed patients meeting the hypothetical screening criterion

**Fig. 4:**
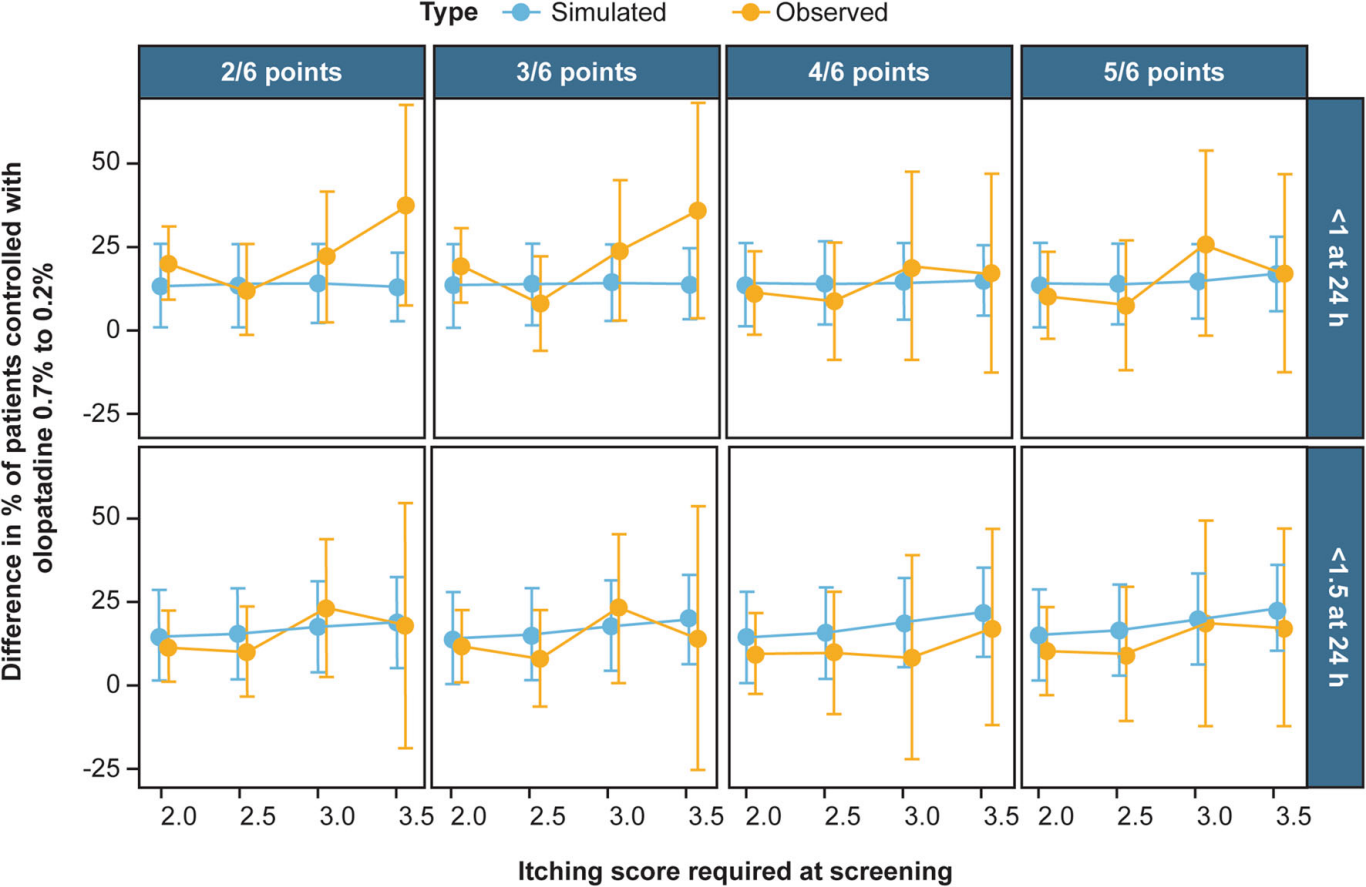
Differences between the percentage of patients adequately controlled (itching score < 1 or < 1.5) at 24 h post-dose when administered olopatadine 0.7% to the percentage of patients at 24 h when administered olopatadine 0.2% based on itching score screening criterion (2/6–5/6 observed baseline points have itching scores ≥ 2.0–3.5). To further compare the difference in the percentage of patients who were controlled on 0.7% over 0.2% was compared by a difference. The difference more clearly shows that the model predicts that the 0.7% dose has a better effect than 0.2%. The 95% confidence interval of the simulations while close to zero, do not include zero, implying this difference is significant at all screening criterion. This significance is very close to α = 5%. However, as with higher baseline itching required and higher number of post-CAC itching observations required for screening, the trends shows a higher level of significance and higher level of relief with the simulated model. On the other hand, the unbalanced observed data show many large confidence intervals, many which do contain zero. These confidence bands are very large, implying for more definitive conclusions, more data is needed. Overall, this figure is suggestive of improved efficacy of the higher 0.7% dose in more severe patients. *CAC* conjunctival allergen challenge

The next question is how well the model describes the data for the higher baseline sensitivity patients. Figure 3 was produced by simulation of the various screening criteria to help answer the question of how many patients experienced itching relief (defined as an average itching score of < 1 or < 1.5) after 24 h of a single 0.2 or 0.7% olopatadine dose. The columns of the figure stratify the study design to have from 2/6 to 5/6 observations needed to be enrolled in the study. The rows are stratified to patients who achieve adequate 24-h relief with the 0.2 and 0.7% olopatadine doses and with two different definitions of adequate relief (mean itching score of all observed times, < 1 at the top and < 1.5 at the bottom). The doses are compared in the first two rows for < 1 itching score having adequate relief. For the 0.7% dose the initial study designs ~ 40% of the population with adequate relief, while the 0.2% has ~ 25% of the population with adequate relief. The shallow slope of curves shows that the 0.7% dose does not change its effect as much as the slightly sharper decline in the 0.2% dose. However, this change in slope difference should be interpreted with caution since it is based on sparse data at the more stringent and may be changed with more data. This trend is also repeated in the bottom two rows, with closer alignment of the data with the simulated trends. This difference in predictive power is likely due to the poorer performance of the no-itching response in the 0.7% dose that was shown in Fig. 2. The data and model is suggestive of more relief from the higher dose in a larger proportion of patients than with the 0.2% dose. Overall Fig. 3 showed that with increasing baseline severity, the percentage of population with relief at 24 h after olopatadine 0.7% was higher than that after olopatadine 0.2% (from 5 to 14% more relief). The percentage of patients who were better controlled on olopatadine 0.7% than olopatadine 0.2% was graphically explored in Fig. 4. The difference more clearly shows that the model predicts that the 0.7% dose has a better effect than 0.2%. The 95% confidence intervals of the simulations while close to zero, do not include zero, implying the model predicts this difference to be significant at all screening criterion, given balanced designs and the same number of subjects. Still, this significance level is very close to α = 5%. Additionally, the simulation shows a higher level of relief, and greater level of significance with more sensitive subjects. On the other hand, the unbalanced observed data show many large confidence intervals, many which do contain zero. Since these confidence bands are very large, more data is needed for more definitive conclusions. At the same time, all of the mean observed differences show at least 5% improvement of olopatadine 0.7% when compared to olopatadine 0.2%, and at most a 38% improvement of 0.7% dose when compared to the 0.2% dose. Additionally the trend of higher responses in higher baselines is maintained. Overall, this figure is suggestive of improved efficacy of the higher 0.7% dose in more severe patients than what would be observed in the 0.2% dose.

In the larger simulated population shown in Fig. 4 the mean difference in 24-h allergy relief shows that olopatadine 0.7% provides itching relief in an additional 10% of the population whose itching could not be controlled with olopatadine 0.2%. As baseline itching scores increases, approximately 25% of the population who could not be relieved with olopatadine 0.2%, had itching relief with olopatadine 0.7%. These differences in the effects between 0.7 and 0.2% olopatadine are shown in Fig. 4.

## Discussion

In order for models to be useful in answering questions outside of the observed data and bridging between modeled data and non-modeled data, the model should be able to (1) predict the outcome of the clinical data used in modeling and (2) predict outcomes of clinical data not used by the model. This model described the observed CAC data itching frequencies well, as expected since it was used to build the model. The observed frequencies of itching scores stratified by time point and treatment were predicted using the model. Additionally, clinical outcomes not used to build the model were predicted (such as average itching score and percentage of patients who had an itching score < 1 at 24 h post-dose), qualifying the overall model. This gives credibility to model-based predictions of 24 h relief with olopatadine at different doses, and allows insight into the possible differences in patient relief at 24 h.

During simulations, the model predicted that a greater proportion of patients had 24 h relief with olopatadine 0.7% than with olopatadine 0.2%, regardless of baseline severity or magnitude of 24 h relief. In all, the simulation stated that olopatadine 0.7% resulted in 25% more 24 h relief than olopatadine 0.2%, which is slightly less than the observed in 38% in the combined studies. Regardless of these generalizations, the proportion of CAC patients who experienced relief with 0.7% olopatadine was higher than those receiving 0.2% olopatadine. Olopatadine 0.7% has an extended duration of action up to 24 h and is particularly useful in patients in whom single dose with a lower strength product is not sufficient to achieve symptomatic relief for a full day. This is confirmed further by increasing effectiveness of olopatadine 0.7% as a function of baseline itching score and severity (Figs. 3, 4). This increasing effectiveness is so substantial that the dose effect was included as a significant covariate of the model. This implies there is an increased effect that is not explained by simply increasing the dose. In addition to the increased effectiveness of 0.7%, the once-daily regimen has several advantages like convenient dosing regimen that may improve patient compliance, reduction in the risk of missed doses and possibly improving treatment outcomes and symptom relief.[18, 20–22] The environmental allergen concentrations can vary throughout the 24 h day-night cycle e.g. patient’s exposure to dust mites is higher during sleep while many plants release pollen during dawn [23]. Thus, the patients may require more frequent dosing with olopatadine 0.2%. The extended duration of action of olopatadine 0.7% over 24 h after administration not only offer significant clinical benefit, but could be a better treatment option for managing symptoms in patients with ocular itching throughout the day or fluctuating itching severities.

These model inferences are based on a few key drivers. First, baseline and vehicle itching scores are assumed to follow the same distribution pattern as what was observed in the prior studies. The distribution of scores is modeled with a differential-odds model to allow the best preservation of the odds observed. This implies that the vehicle, baseline and other covariates affects the categories unequally, implying a more complex relationship than a simple proportional odds model [19]. Furthermore, the only covariates that are unfixed are the 2.5 and 3 scores for the vehicle effect and the 2 or 2.5 scores for the drug effect. This could imply that scores higher than these break-points are not as likely as proportional odds would describe, rather the itching scores are lower than a simple proportional odds model would describe for either the vehicle or drug effect. Additionally, this change-point starts at 2.5 for the vehicle effect, and 2 for the drug effect, also showing that drug itching scores are lower than vehicle itching scores.

Results from a study conducted in a rabbit model showed that 0.77% olopatadine hydrochloride ophthalmic solution resulted in a higher and prolonged olopatadine concentration in the conjunctiva compared to the 0.2% olopatadine ophthalmic solution. While this could explain the prolonged 0.77% effect, the effect observed in this model is based on theoretical drug leaving the effect system. This does not imply that the observed “concentration” nor half-life of the KPD model relate to the rabbit ocular concentrations (when comparing the human half-life to the half-life observed in a rabbit study). Likely any differences in concentrations in rabbits and the theoretical concentrations imply both a down-stream itching response, and an indirect effect model [24]. Furthermore, the olopatadine 0.7% effect is higher than expected given the effects of the other drug concentrations. The last key driver of the model is the baseline effect. Higher baselines lead to more dramatic outcomes. These inferences are still useful to apply this model to look at special populations, such as highly sensitive allergy sufferers.

The model-based approach is a good way to test outcomes of sensitive allergy sufferers without having to run a full-blown environmental clinical trial, or a CAC trial enrolling higher baseline itching subjects. Often environmental trials can fail based on pollen conditions in the day that the trial was run, and the patients selected. CAC trials overcome this hurdle, but may not enroll the breadth of allergy sufferers, possibly missing many of the highly sensitive allergy sufferers. For this reason, a model-based analysis is an appropriate way to characterize the itching response of the highly sensitive allergy sufferers where itching population, as long as the model qualifies in predicting clinical outcomes.

## Conclusion

This simulated study predicted the patients with high itching scores in CAC trials who would require more antihistamine control than that provided by olopatadine 0.2%. This simulated study reconfirms the outcomes of the 2 CAC studies that a greater percent of patients will be controlled after 24 h of dosing with olopatadine 0.7% than olopatadine 0.2%. Although effectiveness data is limited in severe allergy sufferers from controlled clinical studies, this modeling based analysis suggests likelihood of a higher itching relief in the severe allergy sufferers from olopatadine 0.7% than olopatadine 0.2%.
